# Strategies to Target ISG15 and USP18 Toward Therapeutic Applications

**DOI:** 10.3389/fchem.2019.00923

**Published:** 2020-01-21

**Authors:** Daniel Jiménez Fernández, Sandra Hess, Klaus-Peter Knobeloch

**Affiliations:** ^1^Faculty of Medicine, Institute of Neuropathology, University of Freiburg, Freiburg, Germany; ^2^Faculty of Biology, University of Freiburg, Freiburg, Germany

**Keywords:** ISG15, USP18, STAT2, ubiquitin, protein–protein interaction, IFN, Immunity, transgenic mice

## Abstract

The interferon (IFN)-stimulated gene product 15 (ISG15) represents an ubiquitin-like protein (Ubl), which in a process termed ISGylation can be covalently linked to target substrates via a cascade of E1, E2, and E3 enzymes. Furthermore, ISG15 exerts functions in its free form both, as an intracellular and as a secreted protein. In agreement with its role as a type I IFN effector, most functions of ISG15 and ISGylation are linked to the anti-pathogenic response. However, also key roles in other cellular processes such as protein translation, cytoskeleton dynamics, exosome secretion, autophagy or genome stability and cancer were described. Ubiquitin-specific protease 18 (USP18) constitutes the major ISG15 specific protease which counteracts ISG15 conjugation. Remarkably, USP18 also functions as a critical negative regulator of the IFN response irrespective of its enzymatic activity. Concordantly, lack of USP18 function causes fatal interferonopathies in humans and mice. The negative regulatory function of USP18 in IFN signaling is regulated by various protein–protein interactions and its stability is controlled via proteasomal degradation. The broad repertoire of physiological functions and regulation of ISG15 and USP18 offers a variety of potential intervention strategies which might be of therapeutic use. Due to the high mutation rates of pathogens which are often species specific and constantly give rise to a variety of immune evasion mechanisms, immune effector systems are under constant evolutionarily pressure. Therefore, it is not surprising that considerable differences in ISG15 with respect to function and sequence exist even among closely related species. Hence, it is essential to thoroughly evaluate the translational potential of results obtained in model organisms especially for therapeutic strategies. This review covers existing and conceptual assay systems to target and identify modulators of ISG15, ISGylation, USP18 function, and protein–protein interactions within this context. Strategies comprise mouse models for translational perspectives, cell-based and biochemical assays as well as chemical probes.

## Introduction

### ISGylation

ISG15 is one of the genes most strongly induced by type I interferon and was the first Ubiquitin-like modifier (Ubl) identified (Blomstrom et al., 1986; Haas et al., 1987). Analogous to ubiquitin, Ubls like ISG15, small ubiquitin-related modifier (SUMO), human leukocyte antigen (HLA)-F adjacent transcript 10 (FAT10) or neural precursor cell expressed, developmentally down-regulated 8 (NEDD8) can be covalently linked to target proteins to alter a variety of biological processes.

ISG15 is composed of two Ubl domains connected by a flexible polypeptide hinge region. Each domain is formed by four β-sheets and a single α-helix (Narasimhan et al., 2005) reminiscent of the ubiquitin structure. The C-terminal tail of ISG15 contains the LRLRGG motif which is essential for the conjugation to target proteins. Like ubiquitin, ISG15 can be covalently attached to lysine residues of target proteins (through the ε-amino group) via the LRLRGG motif (Loeb and Haas, 1992).

**Figure 1:**
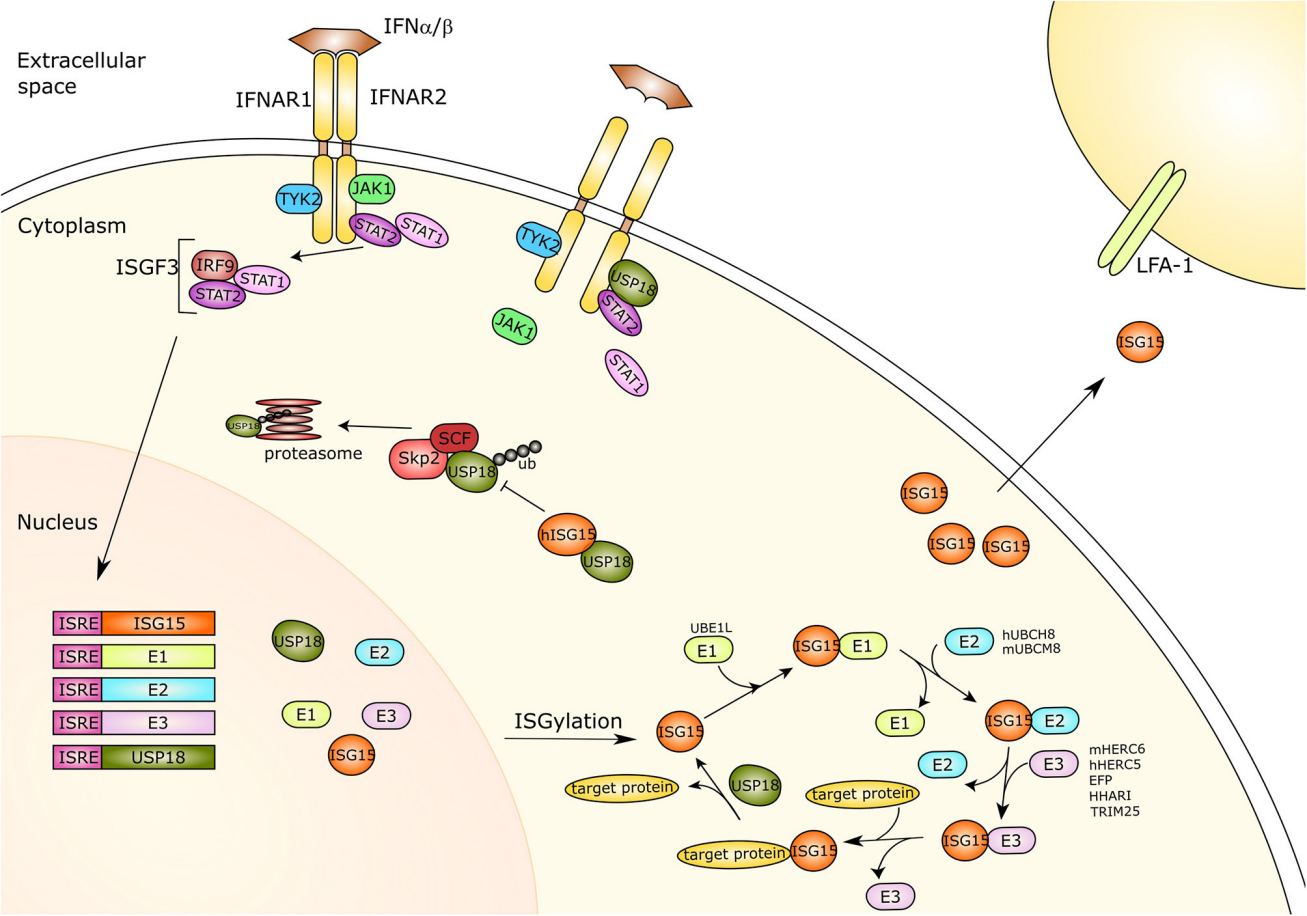
Type-I interferon signaling and ISG15. Type I interferon (IFN) binds its receptor causing the dimerization of the two subunits IFNAR1 and IFNAR2 and thus the activation of the JAK-STAT pathway. The receptor associated kinases TYK2 and JAK1 induce recruitment and phosphorylation of STAT1 and STAT2. The phosphorylated proteins translocate to the nucleus and together with IRF9 form a trimer called ISGF3. This trimer acts as a transcriptional activator and is capable of binding to the ISRE of IFN response genes activating their expression. ISG15 and its three conjugating enzymes E1-activating enzyme (UBE1L), E2-conjugating enzyme (hUBCH8, mUBCM8) and E3 ligases (hHERC5/mHERC6, EFP, HHARI, TRIM25), as well as the ISG15 protease USP18 are all IFN-response genes. ISG15 is linked to target proteins via its conjugation system, which is counteracted by USP18 protease activity. Moreover, free ISG15 can act as a cytokine binding to LFA-1, subsequently inducing IFN-γ secretion by natural killer cells and T lymphocytes. Furthermore, USP18 also plays an important role as a negative regulator of IFN type I signaling. USP18 can interact with IFNAR2 and STAT2, competing with JAK1 for receptor binding and thus inhibiting signal transduction. The SCF^Skp2^ complex binds USP18 by mediating its poly-ubiquitination and proteasomal degradation, which is inhibited by ISG15 in human cells only. IFNAR, Interferon alpha/beta receptor; Tyk2, Tyrosine kinase 2; JAK1, Janus Kinase 1; STAT1/2, Signal transducer and activator of transcription 1/2; IRF9, Interferon regulatory factor 9; ISGF3, Interferon-stimulated gene factor 3; ISRE, Interferon-sensitive response element; ISG15, IFN-response gene 15; UBE1L, Ubiquitin-activating enzyme E1 homolog; h, human; hUBCH8, Ubiquitin/ISG15-conjugating enzyme E2 L6 in human; m, mouse; mUBCM8, Ubiquitin/ISG15-conjugating enzyme E2 L6 in mouse; mHERC6, E3 ISG15-protein ligase HERC6 in mouse; hHERC5, E3 ISG15-protein ligase HERC5 in mouse; EFP, Estrogen-responsive finger protein; HHARI, Human homolog of Drosophila ariadne-1; TRIM25, Tripartite motif-containing protein 25; USP18, Ubiquitin-specific protease 18; LFA-1, Lymphocyte function-associated antigen 1; ub, ubiquitin; SCF, Skp, cullin, F-box protein; Skp2, S-phase kinase-associated protein 2.

Analogous to the ubiquitin conjugation system, ISGylation is mediated by the consecutive action of a three-step catalytic cascade, where all the enzymes are induced by type I IFNs (Figure 1). E1-activating enzymes bind to Ub (or ISG15) and, mediated by ATP-Mg^+2^, form a complex that catalyzes Ub (or ISG15) C-terminal acyl adenylation (Tokgoz et al., 2006). Subsequently, a catalytic cysteine on the E1 enzyme interacts with the ubiquitin-AMP or ISG15-AMP complex undergoing acyl substitution that leads to thioester bond formation and the release of an AMP group. After that, through a transthiolation reaction, an E2 cysteine residue replaces the E1 enzyme. E2-conjugating enzymes catalyze the isopeptide bond formation but also contribute to substrate specificity. E3-ligase enzymes bind the E2-ubiquitin thioester, recognize the protein substrate and catalyze the transfer of ubiquitin or ISG15 from the E2 enzyme to the target protein (Zhang and Zhang, 2011).

In sharp contrast to ubiquitin, which is highly conserved among different species, the amino acid (AA) composition of ISG15 and its effector functions can differ substantially among species. ISG15 has only been identified in vertebrates, and murine ISG15 and its human counterpart share only 64% homology and 76% similarity on the AA level. This is most likely caused by high evolutionary pressure on anti-pathogenic immune effector functions which need to adapt to immune evasion mechanisms from rapidly mutating pathogens.

One of the features that substantially differ in the ISGylation mechanisms between murine and human ISG15 is the use of certain enzymes. The E1 ubiquitin-activating enzyme E1 homolog (UBE1L/UBA7) is a common enzyme for human and mouse in the ISG15 system (Kim et al., 2006), whereas the E1 counterparts for ubiquitin are ubiquitin-like modifier activating enzyme 1 (UBA1) and ubiquitin-like modifier activating enzyme 6 (UBA6) (Pelzer et al., 2007). Ubiquitin/ISG15-conjugating enzyme E2 L6 (UBCH8) and UBCM8 represent the human and murine E2 conjugating enzymes in ISGylation, respectively. Both share only 76% AA identity, whereas E2 conjugating enzymes for other ubl systems show 95–100% identity (Kim et al., 2004). UBCH8 also interacts with the E1-activating enzyme from the Ub conjugation system which indicates an overlap of both conjugation systems at the level of the E2 enzyme (Zhao et al., 2004). However, the enzyme ubiquitin-conjugating enzyme E2 L3 (UBE2L3/UBCH7) represents the dominant conjugating enzyme in ubiquitination as the K_M_ values uncover a 36-fold higher affinity of UBE1L to UBCH7 as compared to UBCH8 (Durfee et al., 2008). Four cellular ISG15 E3 ligases have been identified so far. Human E3 ISG15–protein ligase HERC5 (HERC5) and the murine counterpart E3 ISG15–protein ligase HERC6 (HERC6) are the dominant E3 ligases in ISGylation that coordinate the conjugation of ISG15 to substrates. Interestingly, both mISG15 and hISG15 can be conjugated either by hHERC5 or mHERC6 (Wong et al., 2006; Ketscher et al., 2012). Furthermore, the E3 ubiquitin-protein ligases estrogen-responsive finger protein (EFP) (Zou and Zhang, 2006), human homolog of Drosophila ariadne-1 (HHARI) (Okumura et al., 2007), and tripartite motif-containing protein 25 (TRIM25) (Park et al., 2016) were also reported to mediate ISGylation.

It was shown that ISGylation can occur in a cotranslational process favoring modification of newly synthesized proteins. As in infected cells mainly viral proteins are translated, ISGylation can interfere with pathogen protein function as shown for capsid assembly of the papilloma virus (Durfee et al., 2010). Furthermore, cellular proteins involved in antiviral defense or export of viral particles were shown to be ISGylated (Perng and Lenschow, 2018).

### USP18 Functions: DeISGylation and Negative Regulation of the IFN Response

Ubiquitination and Ubl-conjugation pathways can be reversed by the action of deubiquitinating enzymes (DUBs). These proteases remove or trim Ub/Ubl residues from target proteins. Most of the endogenous proteases from the USP family recognize and deconjugate ubiquitin. However, a small group of proteins from the USP family have been reported to show cross-reactivity and deconjugate ISG15 and ubiquitin, as is the case for USP2, USP5, USP13, USP14, and USP21 (Catic et al., 2007; Ye et al., 2011; Basters et al., 2017).

In addition, many viruses and bacteria have evolved ways to revoke ISGylation as an immune evasion mechanism. Examples of these viral ISG15 proteases were found in the Middle East respiratory syndrome coronavirus (MERS-CoV) (Mielech et al., 2014); Crimean-Congo hemorrhagic fever virus (CCHFV) (Frias-Staheli et al., 2007) or severe acute respiratory syndrome coronavirus (SARS-CoV) (Bekes et al., 2016). They all encode papain-like proteases (PLPs) that impair the host innate immune response.

In contrast to cross-reactive isopeptidases, USP18 is an endogenous ISG15-specific protease that shows no reactivity toward ubiquitin (Malakhov et al., 2002; Basters et al., 2014; Ronau et al., 2016) and it represents the major ISG15 isopeptidase *in vivo* (Ketscher et al., 2012). In order to define the structural function relationship for this specificity, Basters et al., identified the molecular determinants by solving the crystal structures of mouse USP18 alone and in complex with mouse ISG15. USP18 specificity toward ISG15 is mediated by a small interaction interface of two defined areas within the USP18 sequence, termed ISG15-binding box1 and box2 (IBB-1 and IBB-2, respectively). IBB-1 interacts through hydrophobic contact with ISG15. In ISG15, the side chain of His149 stablizes π-π stacking contact to the aromatic AA Trp121. The IBB1 region, which comprises the USP18 residues Ala138, Leu142, and His251, forms a hydrophobic pocket that specifically accommodates the bulky aromatic side chains of ISG15. Furthermore, the side chains of Pro128 (ISG15) and Leu142 (USP18) contribute to further stability. Of note, replacement of the USP18 residues corresponding to the IBB-1 region, by the homologous residues of the ubiquitin specific protease USP7, resulted in lower affinity toward ISG15. Within the IBB-2 region, the USP18 residues Thr262 and Gln259 interact with the ISG15 residues Gln114, His116, and Gln119 through hydrogen bonds. Likewise, replacement of the USP18 residues corresponding to the IBB-2 region, by the homologous residues of the ubiquitin specific protease USP7, resulted in lower affinity toward ISG15. Moreover, only the ISG15 C-terminal domain (AA residues 77-155) is necessary and sufficient for USP18 binding and activation. Structural data demonstrated that only the ISG15 C-terminal but not the N-terminal UBL domain binds USP18. *In vitro* assays revealed that USP18 cleaved the ISG15 C-terminal domain as effectively as it cleaved full-length ISG15 (Basters et al., 2017).

Independent of its deconjugating activity, USP18 binds to the IFN-α/β receptor 2 (IFNAR2) complex, where it competes with Janus kinase 1 (JAK1), and thereby negatively regulates type I IFN signaling (Malakhova et al., 2006). Remarkably, USP18 requires Signal transducer and activator of transcription 2 (STAT2) for exerting its inhibitory effect on IFN signaling and IFN-stimulated gene expression (Arimoto et al., 2017) (Figure 1). In humans, binding of free ISG15 prevents proteasomal degradation of USP18 by the S-phase kinase-associated protein 2 (SKP2) and thus is critical to ensure negative regulation of IFN-α/β immunity by stabilizing USP18 (Tokarz et al., 2004; Zhang et al., 2015). However, murine ISG15 appears not to influence the stability of mouse USP18 or IFNAR signaling underlining species specific peculiarities (Knobeloch et al., 2005; Osiak et al., 2005; Zhang et al., 2015).

### ISG15 as a Secreted Protein

ISG15 in its unconjugated form has been reported to be released from cells exerting cytokine like activity. Although ISG15 does not have a leader signal sequence to direct its secretion, it has been shown that certain cell types are capable of releasing ISG15 to the extracellular space. Such cell types are epithelial-derived cell lines, fibroblasts, monocytes, neutrophils and lymphocytes (Knight and Cordova, 1991; Bogunovic et al., 2012; Sun et al., 2016). Extracellular ISG15 has been detected in the media of cells as well as in the serum of patients treated with IFN-α/β (D'Cunha et al., 1996). Early work suggested that secreted ISG15 elicits IFN-γ secretion from lymphocytes (Recht et al., 1991). Bacillus Calmette–Guérin (BCG) can also induce IFN-γ secretion from control peripheral blood mononuclear cells (PBMCs) when stimulated with recombinant human ISG15 (Bogunovic et al., 2012). In normal control patients, extracellular interleukin (IL)-12 played a synergistic role with ISG15 stimulating the release of IFN-γ and IL-10. Both, natural killer (NK) cells and T lymphocytes secreted IFN-γ in response to IL-12 and ISG15 (Bogunovic et al., 2012). However, IFN-γ secretion was not detected in PBMCs from ISG15-deficient patients and that defect leads to susceptibility to mycobacterial disease and autoinflammation (Bogunovic et al., 2012).

Recently, the adhesion molecule leukocyte function associated antigen-1 (LFA-1) has been identified as the receptor for extracellular ISG15 (Swaim et al., 2017) (Figure 1). To identify this receptor, ISG15 ubiquitin-activated interaction trap (UBAIT) was employed (O'Connor et al., 2015).

## Principal Strategies to Regulate the ISG15 Conjugation System

Many researchers have focused their efforts on the study and characterization of the ubiquitin system (Hershko and Ciechanover, 1998). The ubiquitin-proteasome system (UPS) represents the main mechanism of protein degradation and the regulation of every step within this mechanism is crucial to prevent several disorders and diseases such as tumor development and progression. A recent example of an existing drug that targets the ubiquitin system is the adenosine sulfamate inhibitor, TAK-243, which inhibits the ubiquitin activation enzyme (E1) (UAE/UBA1) (Hyer et al., 2018). TAK-243 has entered phase I trial studies for the treatment of patients with relapsed or refractory acute myeloid leukemia, refractory myelodysplastic syndrome or chronic myelomonocytic leukemia (NCT03816319).

Two other compounds have been described to target UAE/UBA1: PYR-41, a cell permeable inhibitor that blocks the catalytic cysteine (Yang et al., 2007) and panepophenanthrin, a fungal product which inhibits ubiquitin thioester formation (Sekizawa et al., 2002). The pyrazolidine compound 4-[4-(5-nitro-furan-2-ylmethylene)-3,5-dioxo-pyrazolidin-1-yl]-benzoic acid ethyl ester was shown to inhibit UBA1. Likewise, the analog drug PYZD-4409, that carries a pyrazolidine pharmacophore, also inhibited UBA1 activation and therefore subsequent transfer of ubiquitin from the E1 to the E2 enzyme. This effect resulted in tumor growth delay in a mouse model of leukemia (Xu et al., 2010b). Besides ubiquitin, a NEDD8 activating enzyme (NAE) inhibitor has been characterized. MLN492 is a nucleotide analog that binds to UBA3/NAE1 (NEDD8 E1 enzyme) and inhibits NAE function in cells and suppresses the growth of human tumor xenografts. This chemical has entered phase II studies with promising results as an anti-cancer drug in acute myelogenous leukemia (AML) or high-grade myelodysplastic syndrome (MDS) (Soucy et al., 2009).

The drug CC0651 works as an allosteric inhibitor of the human ubiquitin-conjugating enzyme E2 R1 (CDC34) (Ceccarelli et al., 2011). Binding of CC0651 to CDC34 causes secondary structural rearrangements preventing the ubiquitin transfer to substrates. In this case, ubiquitin thioester formation is not compromised and neither is the interaction with E1 and E3 enzymes. Hence, it shows the importance of the E2 enzymatic step as a regulation point in the process of ubiquitination. In addition, NSC697923 has been developed to target ubiquitin-conjugating enzyme variant (UBC13-UEV1A), an E2-conjugating enzyme, blocking ubiquitin transfer to the substrate (Pulvino et al., 2012). Furthermore, BAY 11-7082 interacts with the ubiquitin-conjugating enzyme E2 N (UBE2N) and modifies the reactive cysteine residue of the E2 enzyme (Strickson et al., 2013).

Analogous to ubiquitin or other UBL modification systems, the cascade of conjugation enzymes comprise targets to affect ISGylation. Likewise, USP18 inhibition (see below) represents a strategy to stabilize ISGylation. Furthermore, the ISG15 cell surface receptor represents a target to modulate ISG15 function. As indicated before, ISG15 also exists as an unconjugated protein and it has been proposed to function as a cytokine (Swaim et al., 2017). The ISG15 cell surface receptor LFA-1 is a heterodimeric complex that comprises two subunits, CD11a/αL and CD18/β2. The CD11a/αL domain forms part of the binding site for both Intercellular Adhesion Molecule 1 (ICAM1) (Shimaoka et al., 2003) and ISG15. However, determinants of the αL domain recognized by ISG15 and ICAM1 are different and biochemically and biologically separable (Swaim et al., 2017). Extracellular ISG15 plays an important role in the secretion of cytokines such as IL-10 or IFNγ. Therefore, ISG15 can be potentially exploited to boost cytokine secretion (Swaim et al., 2017). In contrast, targeting LFA-1 receptor or specifically one of the two heterodimers that form the receptor would conceptually lessen the impact of extracellular ISG15 activity by blocking its receptor.

## USP18 Activity Assays

Activity-based probes (ABPs) are a helpful tool to study the activity of DUBs and Ubl-specific proteases. One of the main advantages of using ABPs over other substrate-based probes is that ABPs covalently attach to the active site of the target protein (Ovaa, 2007; Verdoes and Verhelst, 2016). Many proteases are secreted in their inactive forms and require post-translational modifications to become active. These modifications can be either irreversible, via proteolysis, or reversible by pH change or protein complex formation (Hewings et al., 2017). The characterization of DUBs in biological processes benefits from ABPs as protein activity is rather important and classical methods such as western blotting or proteomics techniques are not suitable to deduce enzyme activity. Furthermore, fluorogenic substrates are a valuable tool for kinetic studies as they are turned over by the target enzyme and as a consequence the resulting fluorescent signal will be proportional to enzyme activity.

**Figure 2:**
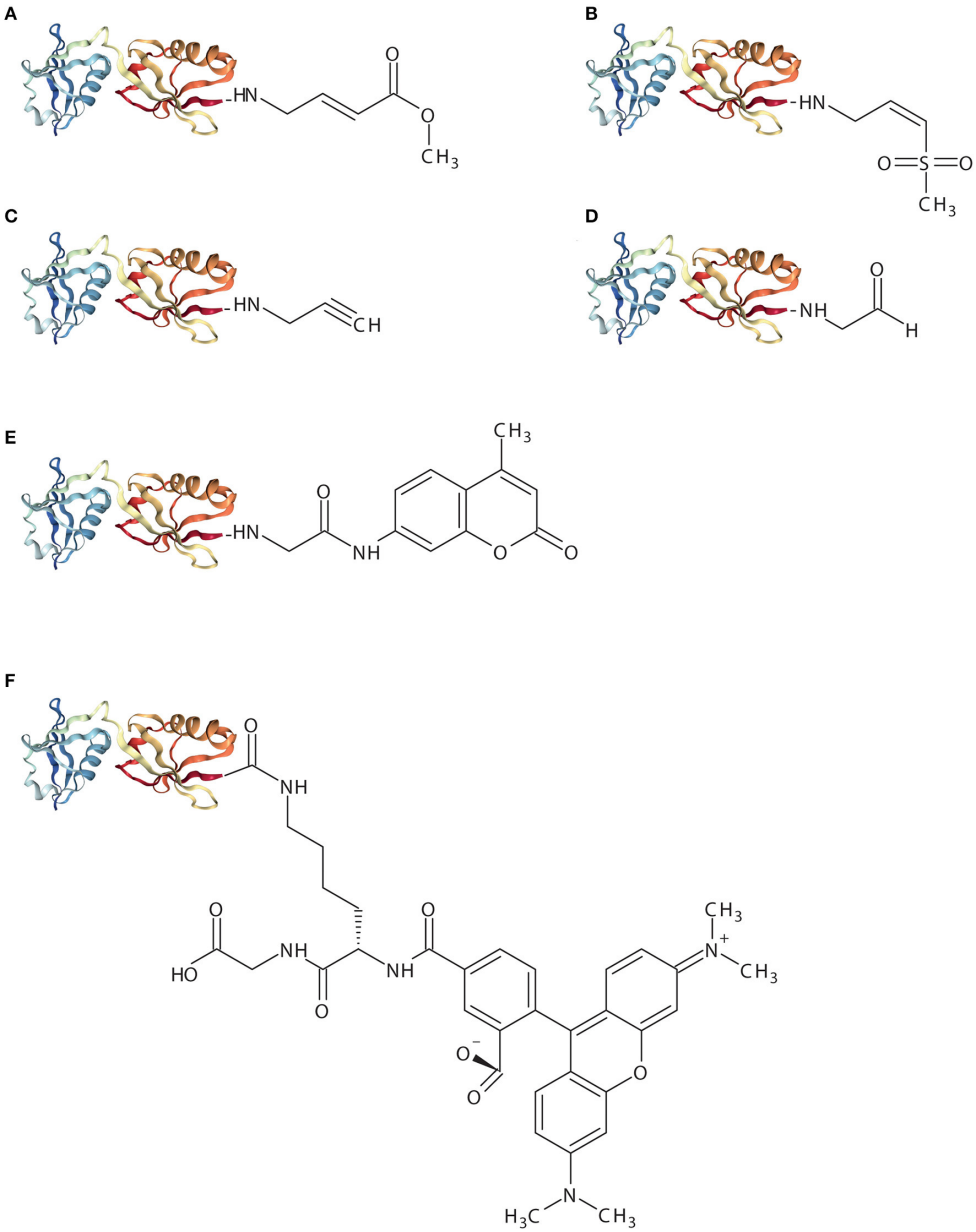
Schematic representation of ISG15-based assay reagents. The X-ray crystal structure represents mouse ISG15 (PDB: 5TLA) and implies ISG15_1−155_. **(A)**, ISG15-VME **(B)**, ISG15-VS **(C)**, ISG15-PRG **(D)**, ISG15-CHO **(E)**, ISG15-AMC **(F)**, ISG15-TAMRA-(5-thioLys)-Gly.

As mentioned before, USP18 specifically deconjugates ISG15 from substrates (Malakhov et al., 2002). Specific ABPs for USP18 proteases have been developed (Ekkebus et al., 2013; Basters et al., 2017). In analogy to ABPs for ubiquitin, these probes have been synthesized by replacing the C-terminal residue of ISG15 with an electrophilic moiety, such as—VS (Vinyl methyl sulfone), VME (Vinyl methyl ester), or PRG (Propargylamide) (Ekkebus et al., 2013; Basters et al., 2017). These probes work as a C-terminal electrophilic trap and they were synthesized with intein chemistry (Hemelaar et al., 2004) (Figure 2). The use of these ISG15 probes represents a valuable tool to evaluate the enzymatic activity of DUBs.

ISG15-VME covalently binds the active-site cysteine via thioether bond forming a covalent complex. The sulfur atom of the active-site cysteine of the DUB interacts with the carbon (β) atom of the VME moiety forming a thiol bond (Boudreaux et al., 2010). ISG15-VME and ISG15-VS form covalent adducts via a Michael-type (1,4) addition. Vinyl sulfone reactions can be performed under physiological conditions (e.g., aqueous media, slightly alkaline pH) which preserves the biological function of the proteins (Morales-Sanfrutos et al., 2010).

ISG15-VS results in the formation of a covalent complex between USP18 and ISG15. The reaction results in a covalent adduct that can be detected by SDS/PAGE as an upwards shift in molecular mass. A similar experiment was conducted making use of the equivalent propargylamide probe, ISG15-PRG, with USP18-ISG15 complex formation. ISG15-PRG forms a vinyl thioether with the DUB active site cysteine residue. The reaction depends on a direct nucleophilic attack on the internal alkyne carbon as a result of the developing carbanion stabilization by the “oxyanion hole” of the active site (Ekkebus et al., 2013; Hewings et al., 2017). Conversely, Ub-VS or Ub-PRG did not react with USP18 demonstrating that USP18 does not exert enzymatic activity toward ubiquitin (Basters et al., 2014, 2017).

The crystal structure of mouse USP18 in complex with mouse ISG15 displayed extensive interaction between the ISG15 C-terminal Ubl domain and the palm and thumb domain of USP18 (Basters et al., 2017). A good example for the use of an ISG15-PRG probe was the validation of the ISG15 C-terminal domain as necessary and sufficient for USP18 binding. Here, either only the C-terminal Ubl domain of ISG15 (ct-ISG15) or full-length ISG15 were fused to -PRG to form the respective ct-ISG15-PRG and ISG15-PRG probes. Both probes reacted with the active site cysteine of USP18 and formed a covalent complex. Furthermore, USP18 cleaved ct-ISG15 as effectively as it cleaved full-length ISG15 from cellular substrates (Basters et al., 2017).

Synthesis of the ISG15 C-terminal Ubl domain was carried out through solid-phase peptide chemistry (SPPS). Briefly, SPPS is an automated synthesis method used for the production of synthetic peptides. This technology allows assembly of a peptide chain through successive reactions of amino acids or derivatives. The activated carboxyl moiety of each incoming amino acid is linked to the α-amino group of the subsequent amino acid. The new α-amino group gets protected by 9-fluorenylmethoxycarbonyl (Fmoc) to avoid unintended peptide bond formation at this site until the incoming amino acid is added to the sequence. In addition, reactive side chains on the amino acids are protected by ester, ether and urethane derivative complex formation during the synthesis of the synthetic peptide (El Oualid et al., 2010). Recently, the synthesis of the N-terminal domain together with the C-terminal domain, comprising the 155 amino acid protein mISG15, has been reported (Xin et al., 2019). Most of the ISG15 functions, especially those related to deISGylation, is attributed to its C-terminal domain. The full length synthesis of mISG15 will elucidate specific roles associated to the N-terminal domain within ISG15.

High-throughput drug screening (HTS) studies in combination with enzymatic assays using ubiquitin-7-Amino-4-methylcoumarin (Ub-amc) have been extensively used for the identification of small molecules inhibiting USP protease activity (Hirayama et al., 2007). Hydrolysis of the fluorophore amc group upon cleavage of the isopeptidase bond by a specific protein results in a quantifiable fluorescence signal. ISG15-amc was used to demonstrate USP18 activity and specificity toward ISG15 (Basters et al., 2012) (Figure 2). Therefore, the ISG15-amc probe can also be used to monitor activity of other deISGylases (e.g., viral ISG15 DUBs).

ISG15-Rhodamine represents a fluorescence polarization (FP) assay reagent where ISG15 is quenched to the green fluorescence rhodamine 110 cationic dye by its C-terminus. The substrate was synthesized from ISG15^C76S^ and 5-carboxytetra-methylrhodamine (TAMRA)-labeled 5-thioLys-Gly dipeptide. The linkage between the C-terminal Gly of ISG15 and a Lys side chain resembles natural ISG15-linked substrates more precisely (Tirat et al., 2005; Geurink et al., 2012; Swatek et al., 2018). Likewise, hydrolysis of the TAMRA-Lys-Gly complex by a specific protein results in a quantifiable fluorescence signal. Hence, incubation of ISG15-TAMRA with USP18 led to a dose-dependent decrease of polarization values (in millipolarization), indicative of proteolytic cleavage of the substrate (Basters et al., 2014) (Figure 2).

The reversible ISG15 aldehyde inhibitor, ISG15-CHO, does not yield a stable adducted enzyme but it still represents a highly specific inhibitor of ISG15-specific isopeptidases (Siklos et al., 2015) (Figure 2). This probe blocks the hydrolysis of poly-ISG15 chains on substrate proteins *in vitro*. However, aldehyde inhibitors suffer metabolic oxidation/reduction modifications and pH-dependent hydrate formation that results in deficient stability and bioavailability. Such drawbacks limit the progress of aldehyde inhibitors to the clinic.

## ISG15- and USP18-Related Diseases

Due to the critical role of the ISG15 system in antimicrobial host defense it is appealing to exploit this endogenous effector system therapeutically. Within this context, human patients lacking functional ISG15 represent valuable subjects of investigation to define physiological and molecular functions. Six patient cases with ISG15 deficiency from three non-related families have been reported (Bogunovic et al., 2012; Zhang et al., 2015).

In mice, ISG15 plays an important role in host response to viral infection. It has been shown to protect from viral-induced lethality using different pathogens (Perng and Lenschow, 2018). However, ISG15-null patients appear not to be more susceptible to viral infections (Bogunovic et al., 2012). Conversely, ISG15 deficient patients even showed enhanced antiviral protection (Speer et al., 2016). Three of the ISG15 deficient patients suffered from seizures and displayed intracranial calcification, which is a common phenotype for patients with Aicardi-Goutières syndrome (AGS) (Zhang et al., 2015). In humans, binding of free ISG15 prevents proteasomal degradation of USP18 by SKP2 (Tokarz et al., 2004) and is critical to ensure negative regulation of IFN-α/β immunity by stabilizing USP18 (Zhang et al., 2015). The three ISG15 deficient individuals showed hyper-responsiveness to type-I IFN stimulation due to the fact that human USP18 stability relies heavily on human ISG15. Thus, in the absence of ISG15 USP18 would no longer be able to function as a negative regulator of type-I IFN signaling (Zhang et al., 2015). The regulatory function of ISG15 to stabilize USP18 is not seen in mice (Speer et al., 2016). Recently, USP18 deficient patients were identified (Meuwissen et al., 2016). These patients' life expectancy is quite short and they die shortly after birth due to massive dysregulation of type-I IFN signaling. Five Pseudo-TORCH syndrome (PTS) patients showed recessive loss-of-function mutations of USP18 leading to severe immune inflammation with calcification and polymicrogyria. USP18 deficient patients represent the first case of a genetic disorder of PTS caused by dysregulation of the response to type I IFNs. This situation makes USP18 an interesting therapeutic target, as USP18 agonists might be a strategy to dampen type-I IFNs overabundance. Alternatively, USP18 antagonists or strategies promoting USP18 degradation could promote the beneficial effect of therapeutic IFNs used in multiple sclerosis, hairy cell leukemia, and melanoma (Meuwissen et al., 2016).

Studies in mice lacking USP18 uncovered a key role of USP18 to maintain microglial quiescence under homeostatic conditions (Goldmann et al., 2015; Schwabenland et al., 2019). USP18 negatively regulates the activation of STAT1 upon interaction with IFNAR2 (Malakhova et al., 2006). Interestingly, this regulatory function is independent from USP18 catalytic activity as it was also observed in knock-in mice (USP18^C61A/C61A^), expressing enzymatically inactive USP18. USP18^C61A/C61A^ mice showed increased resistance against virus infections, but in contrast to USP18^−/−^ mice, USP18^C61A/C61A^ knock-in mice did not display fatal IFN hypersensitivity, brain injury or increased lethality (Ketscher et al., 2015). Based on the analysis of USP18^C61A/C61A^ mice, selective inhibition of USP18 proteolytic activity might be used as an antiviral strategy.

## Identification of ISG15 Substrates

To gain further insight into ISG15 targets, it would be interesting to define ISG15 modified proteins on a proteome wide base (ISGylome) to identify specific ISG15 modifications sites, and to uncover common principles of ISG15 modification. Several proteomics studies have identified hundreds of cellular but also viral substrates (Giannakopoulos et al., 2005; Zhao et al., 2005). In these studies, ISG15-modified proteins were purified from IFN-β-treated cells by using both affinity selection and mass spectroscopy (MS-MS) to identify ISG15 target proteins. Later, a new study compared the proteomes of ISG15^+/+^ and ISG15^−/−^ bone marrow derived macrophages (BMDMs) upon vaccinia virus (VACV) infection (Baldanta et al., 2017). Here, they evaluated the presence of ISGylated proteins in total extracts from ISG15^+/+^ and ISG15^−/−^ BMDMs that were left untreated or treated with IFN or VACV. The results indicated mitochondrial dysfunction and oxidative phosphorylation (OXPHOS) in ISG15^−/−^ mice (Baldanta et al., 2017). Further analysis of the ISG15 target proteins will shed light on the different functions of ISG15 in the innate immune system.

Recently, Swatek et al. (2018) have elucidated a mechanism to identify virus-induced modified proteins upon foot-and-mouth disease (FMD). The viral leader protease, Lb^pro^, mainly targeted ISG15 showing high activity and specificity for ISG15 over other Ubl proteins. Lb^pro^ cleaves the peptide bond preceding the ISG15 C-terminal GlyGly motif; consequently cleaved ISG15 can no longer be reconjugated, leading to shut down of the ISG15 modification system. Unlike Lb^pro^, USP18-mediated ISG15 cleavage leads to ISG15 recycling since USP18 cleaves the isopeptide linkage after the C-terminal GlyGly motif and ISG15 remains competent for reconjugation. Lb^pro^ activity has been quantified using fluorescence polarization assay reagents (Swatek et al., 2018). Importantly, Lb^pro^ represents a new tool to uncover virus-induced GlyGly remnants on substrate proteins using an anti-GlyGly antibody already used for ubiquitin MS-MS research.

Peptide enrichment by immunoprecipitation (IP) technology is developed to quantitatively profile modification sites in cellular proteins. Ubiquitin as well as other Ubls can be covalently linked to lysine residues of target proteins. The bead-conjugated Lys-ε-GG antibody specifically recognizes the GlyGly remnant left after trypsin digestion of modified proteins (Udeshi et al., 2013). Enrichment upon Lys-ε-GG antibody IP coupled with liquid chromatography (LC) tandem MS-MS analysis leads to the identification of a substantial number of proteins modified with ubiquitin or Ubls. Ubiquitin and some Ubls share a common diglycine adduct upon digestion with trypsin. The identification of ISG15-modified sequences would represent a valuable tool to characterize new molecular pathways in situations of homeostasis or disease-related conditions. Recently, the endogenous *in vivo* ISGylome in mouse liver, following *Listeria* infection has been mapped. In this study, authors employed Lys-ε-GG antibody IP in wildtype and ISG15^−/−^ mice followed by LC tandem MS-MS analysis. Comparison of the datasets allowed to identify and distinguish ISGylated sites from ubiquitin sites *in vivo* (Zhang et al., 2019). Similar approaches have already been used in several cell systems to identify different post-translational modifications such as, phosphorylation (Rush et al., 2005), ubiquitination (Xu et al., 2010a; Kim et al., 2011), acetylation (Weinert et al., 2011; Kori et al., 2017), methylation and SUMOylation (Impens et al., 2014; Lamoliatte et al., 2017).

## Novel Technologies to Target USP18

Proteolysis Targeting Chimeras (PROTACs) or degronimids are reagents that recruit a protein of interest to a specific ubiquitin E3 ligase. The E3 ligase induces its ubiquitination followed by subsequent degradation by the proteasome. These probes are bifunctional small molecules that combine a target-binding warhead and E3 ubiquitin ligase-recruiting moiety by a chemical linker (Sakamoto et al., 2001; Stanton et al., 2018). This drug discovery strategy differs from classical methods that focus on targeting the protein of interest by specific inhibitors or its receptor ligands. Interestingly, ARV-110 represents the first oral PROTACs drug that has been approved by the FDA for the treatment of patients with metastatic castration-resistant prostate cancer (NCT03888612). PROTACS targeting USP18 might represent an interesting approach to specifically degrade USP18 and thus enhance type I IFN signaling.

Beside direct destabilization, targeting the interaction of USP18 with important proteins such as STAT2 or ISG15 might constitute an option to interfere with its function.

A sophisticated technique to directly study protein–protein interaction within a cellular context is the BRET (Bioluminescence Resonance Energy Transfer) assay where dipole-dipole energy is transferred from a luciferase to a fluorophore. For the successful energy transfer, the excitation spectrum of the acceptor fluorophore has to overlap with the bioluminescence spectrum generated by the luciferase (Ciruela, 2008). Similar to fluorescence resonance energy transfer (FRET), this transfer is dependent on close proximity (<10 nm) between the donor/acceptor pair (Wu and Brand, 1994; Pfleger and Eidne, 2006). Thus, genetic fusion of this system to proteins of interest can be used to measure their protein interaction. This is achieved by creating fusion constructs of the donor luciferase with one protein of interest and the acceptor with a second protein of interest.

In the case of NanoBRET^™^, NanoLuc^®^ represents a genetically modified luciferase, originating from the deep sea shrimp *Oplophorus gracilirostris* that acts as donor. Genetic engineering and the use of a novel coelenterazine derivate (Furimazine) resulted in a brighter luminescence, with a narrower spectrum and higher protein stability compared to the traditional RLuc (Hall et al., 2012). For the acceptor fusion, a red-emitting fluorophore is linked to the HaloTag^®^ protein (Machleidt et al., 2015). HaloTag^®^ is a modified bacterial haloalkane dehydrogenase which can covalently bind fluorescent dyes or other molecules of interest through a chloroalkane linker allowing for tailoring the Tag for each individual experimental setup (Los et al., 2008; Machleidt et al., 2015).

## Discussion and Future Perspectives

Multiple strategies have been proposed to modulate ISG15 function in the immune system. The endogenous ISG15-specific protease, USP18, shows no reactivity toward ubiquitin (Malakhov et al., 2002; Basters et al., 2014; Ronau et al., 2016) and represents the major ISG15 isopeptidase *in vivo*.

How the negative regulation of type I IFN by USP18 is precisely mediated is only starting to become clearer in recent years, and additional proteins and factors involved await discovery. Moreover, it is unknown whether the enzymatic and the non-enzymatic functions of USP18 are really exerted in an independent manner or can influence each other. Protein–protein interaction assays such as NanoBRET^™^ represent a technique to monitor protein–protein interactions involving USP18, STAT2, IFNAR, and ISG15 more closely. As a cell-based assay, it appears to be well-suited to analyze the interaction of two proteins of interest under physiological conditions nicely complementing biochemical assays.

Traditional strategies to inhibit enzyme activity have focused on the development and synthesis of small molecules that bind to the active side of a protein of interest in order to decrease its activity. However, besides the classical biochemical protease assay where recombinant USP18 is used in combination with ISG15-TAMRA (Basters et al., 2014), a screening system suitable to monitor direct USP18-ISG15 binding could be helpful.

In the classical approach targeting a protease, a small molecule needs to show activity toward the enzymatic activity either by blocking the binding pocket or allosteric mechanisms. This highly specific requirement often makes it difficult to identify suitable compounds even in HTS approaches. However, libraries of covalent inhibitors could be beneficial for chemical screens to identify new compounds.

PROTACs represent a very elegant strategy to target difficult druggable proteins by selectively targeting their degradation through the proteasome (Sakamoto et al., 2001; Stanton et al., 2018). Target proteins with low affinities for PROTACs can still be targeted and further degraded as long as the formation of the PROTACs complex generates sufficient protein–protein interactions between the target protein and the E3 ligase (Bondeson et al., 2018). It is therefore important to consider other parameters such as an adequate linker or a specific E3 ligase on top of traditional inhibitor specificity. As described above, the ISG15-TAMRA/recombinant USP18 assay represents a HTS compatible system to identify inhibitors of USP18 enzymatic activity. Compounds identified in such a screen are expected to bind USP18 with high affinity or even covalently and thus should also constitute interesting building blocks for PROTACs aiming to degrade the entire USP18 protein. Rather than only stabilizing ISGylation PROTACS directed against USP18 would be expected to also boost the type I IFN response as USP18 can no longer exert its negative regulatory function at the IFNAR. Thus, targeted degradation of USP18 might at least conceptually be a strategy to enhance the therapeutic use of type I IFNs in antiviral, antineoblastic and autoimmune applications. Currently, there are several mouse models to study the function of ISG15: ISG15^−/−^ (Osiak et al., 2005), UbE1L^−/−^ (Kim et al., 2006), USP18^−/−^ (Ritchie et al., 2002) and USP18^C61A/C61A^ mice (Ketscher et al., 2015). ISG15^−/−^ mice lack both free ISG15 and ISG15 conjugates. UbE1L^−/−^ mice show higher basal and inducible levels of free ISG15; however, these mice lack ISG15 conjugates. USP18^−/−^ mice present higher basal and inducible levels of ISG15 modified proteins. The USP18^C61A/C61A^ mouse expresses a catalytic inactive form of USP18 and thus mimics the scenario of USP18 protease activity repression via a small molecule inhibitor. This mouse model shows enhanced ISGylation levels because of the USP18 protease inactivation whereas they do not show apparent phenotypic alterations (Ketscher et al., 2015). Furthermore, no abnormalities were identified in USP18^C61A/C61A^ mice backcrossed to C57BL/6, a genetic background, in which USP18^−/−^ mice display malformations that leads to embryonic lethality around embryonic day 15.5 (E15.5) (Ketscher et al., 2015). The use of a humanized ISG15 mouse model where murine ISG15 is replaced by its human counterpart would represent an interesting *in vivo* model to study species-specific differences in ISG15 with respect to substrate recognition and antipathogenic activity. It is known that E1, E2, and E3 enzymes can be exchanged between mouse and human and that mUSP18 can efficiently deconjugate ISGylated substrates derived from IFN treated human cells (Ketscher et al., 2012). Therefore, this mouse model would shed light on how murine and human ISG15 can target proteins upon infection with different pathogens.

In conclusion, the use of all these different mouse models in combination with enrichment of modified peptides by IP and further MS-MS technology, developed to quantitatively profile modification sites in cellular proteins, represent valuable tools to unravel the ISGylome in situations of homeostasis or disease-related conditions.

## Author Contributions

DJ and K-PK discussed and developed the concept of the review. DJ, SH, and K-PK edited the manuscript. DJ and K-PK wrote the manuscript.

### Conflict of Interest

The authors declare that the research was conducted in the absence of any commercial or financial relationships that could be construed as a potential conflict of interest.

## Abbreviations

IFN: Interferon
ISG15: IFN-stimulated gene product 15
Ubl: Ubiquitin-like protein
USP18: Ubiquitin-specific protease 18
STAT2: Signal transducer and activator of transcription 2
ABPs: Activity-based probes
SUMO: Small ubiquitin-related modifier
FAT10: Human leukocyte antigen (HLA)-F adjacent transcript 10
NEDD8: Neural precursor cell expressed, developmentally down-regulated 8
AA: Amino acid
UBE1L: Ubiquitin-activating enzyme E1 homologue
UBA1: Ubiquitin-like modifier activating enzyme 1
UBA6: Ubiquitin-like modifier activating enzyme 6
UBCH8: Ubiquitin/ISG15-conjugating enzyme E2 L6 in human
UBCM8: Ubiquitin/ISG15-conjugating enzyme E2 L6 in mouse
UBE2L3/UBCH7: Ubiquitin-conjugating enzyme E2 L3
HERC5: E3 ISG15-protein ligase HERC5
HERC6: E3 ISG15-protein ligase HERC6
h: Human
m: Murine
EFP: Estrogen-responsive finger protein
HHARI: Human homolog of Drosophila ariadne-1
TRIM25: Tripartite motif-containing protein 25
DUB: Deubiquitinating enzyme
MERS-CoV: Middle East respiratory syndrome coronavirus
CCHFV: Crimean-Congo hemorrhagic fever virus
SARS-CoV: Severe acute respiratory syndrome coronavirus
PLP: Papain-like protease
IBB1: ISG15-binding box1
IBB2: ISG15-binding box2
IFNAR2: IFN-α/β receptor 2
JAK1: Janus kinase 1
SKP2: S-phase kinase-associated protein 2
BCG: Bacillus Calmette–Guérin
PBMCs: Peripheral blood mononuclear cells
IL: Interleukin
NK: Natural killer
LFA-1: leukocyte function associated antigen-1
UBAIT: Ubiquitin-activated interaction trap
UPS: Ubiquitin-proteasome system
UAE: Ubiquitin activation enzyme (E1)
CDC34: Human ubiquitin-conjugating enzyme E2 R1
UBC13-UEV1A: Ubiquitin-conjugating enzyme variant
UBE2N: Ubiquitin-conjugating enzyme E2 N
ICAM1: Intercellular Adhesion Molecule 1
VS: Vinyl methyl sulfone
VME: Vinyl methyl ester
PRG: Propargylamide
ct-ISG15: C-terminal Ubl domain of ISG15
SPPS: Solid-phase peptide chemistry
FMOC: 9-fluorenylmethoxycarbonyl
HTS: High-throughput drug screening
amc: 7-Amino-4-methylcoumarin
FP: Fluorescence polarization
TAMRA: 5-carboxytetra-methylrhodamine
CHO: Aldehyde
AGS: Aicardi-Goutières syndrome
PTS: Pseudo-TORCH syndrome
MS: Mass spectroscopy
BMDMs: Bone marrow derived macrophages
VACV: Vaccinia virus
OXPHOS: Oxidative phosphorylation
FMD: Foot-and-mouth disease
IP: Immunoprecipitation
LC: Liquid chromatography
PROTACs: Proteolysis Targeting Chimeras
BRET: Bioluminescence Resonance Energy Transfer
FRET: Fluorescence resonance energy transfer.
